# Effect of Coating Thickness on Abrasion and Cutting Performance of NCD-Coated Ball Endmills on Graphite Machining

**DOI:** 10.3390/mi14030664

**Published:** 2023-03-16

**Authors:** Hyeonhwa Lee, Jinsoo Kim, Sungcheul Lee, Jongeun Park, Jeongyeon Park, Jongsu Kim

**Affiliations:** 1 Molding & Metal Forming R&D Department, Korea Institute of Industrial Technology, Bucheon 14441, Republic of Korea; 2 Department of Ultra-Precision Machines and Systems, Korea Institute of Machinery & Material, Daejeon 34103, Republic of Korea; 3 Department of Mechanical Engineering, The State University of New York, Korea (SUNY Korea), Incheon 21985, Republic of Korea

**Keywords:** nano-diamond coating, coating thickness, nano-aggregated structure, coating abrasion mechanism, machined surface roughness, graphite machining

## Abstract

Nano-crystalline diamond (NCD) coating to improve the performance of cutting tools, as the coating thickness varies, the cutting performance and lifespan of the tool varies because the radius of its cutting edge and coating surface roughness are altered. Therefore, an in-depth analysis on the impact of the variations in coating thickness on the cutting tool abrasion and quality of machined surface is necessary. In this study, two NCD ball endmills were coated with 8 and 12 μm thicknesses, and the tool abrasion and roughness of the machined plane were observed after milling. Furthermore, the morphology of the coated surface and abrased cutting edge were observed using a 3D confocal microscope. Consequently, we observed that individual nodules were formed on the continuous aggregates as the coating thickness increased, which increased the coated surface roughness. The two damage modes of the aggregation determined the dominant abrasion that occurred on the cutting edges of both types of coating thicknesses. Delamination and crater wear caused a sharp increase in the roughness of the machined surface. In summary, the increase in coating thickness delayed the delamination of the coating but increased the roughness of the cutting edge, which reduced the machined surface roughness.

## 1. Introduction

Diamond coating in the field of mechanical cutting is mainly applied in cutting tools for highly abrasive materials, such as composites, aluminum alloys, high-hardness graphite, and sintered ceramics. In addition to being chemically inactive, diamond has low friction, high hardness, and outstanding abrasion durability. Therefore, it is used for the purpose of improving the lifespan and cutting performance of cutting tools. Furthermore, it facilitates the nucleation of tungsten carbide (WC) substrates, due to similar thermal expansion coefficients and bonding characteristics. Hence, it is advantageous for strengthening the performance of carbide tools [1,2]. Diamond coating by chemical vapor deposition (CVD) is commercially available, and the type of coating is distinguished by the size of diamond particles. Among the various types of coatings, microcrystalline diamond (MCD) coatings consisting of cone-shaped particles that are 3–5 μm in size have relatively strong adhesion strength to the base material. Therefore, they increase the tool’s lifespan by delaying delamination. However, their coating surface roughness and cutting-edge radius increases, which are unfavorable for precision cutting. Nano-crystalline diamond (NCD) coating, wherein tens of nanoparticles are combined, shows opposite properties to MCD coating. While the delamination of NCD coating is easier than that of MCD coating, and the lifespan of NCD coated tools are shorter than those of MCD coated tools, the geometric error of the tool is, relatively, smaller. Hence, NCD coated tools are, relatively, more advantageous in precision cutting [3,4]. Thus, many studies have attempted to improve the lifespan of NCD-coated tools [4,5,6,7,8,9]. The coating thickness changes the cutting-edge radius and coating surface, which affect the geometric precision of the tool. Therefore, it is a significant factor in the performance assessment of the coated tool. According to Qin [5], there is a lack of research that analyzes the impact of the coating thickness on the lifespan and cutting performance of the tools. Moreover, recent studies are primarily focused on developing new coating materials and improving the process [4,7,8,9].

Qin [5] analytically and experimentally studied the impact of the coating thickness, and performed external diameter turning after coating the insert with NCD using the high-power micro-wave plasma-assisted CVD method. It was reported that it also leads to higher delamination resistance and longer tool lifespan, even though increasing the coating thickness increases the residual stress on the coating-substrate interface. In addition, the radius of the edge increases as the coating grows thicker, but the impact on the cutting load varies according to the processing condition. Kanda [6] assessed the abrasion of various cutting tools that were diamond coated and reported that an increased film thickness favored the tool’s lifespan. However, the author described that some issues need to be addressed to realize a widespread supply of diamond-coated cutting tools, including the increased roundness of the cutting edge due to film thickness and reduced values of coating surface roughness and the transverse fracture strength. Bian [4] experimentally analyzed tool performance, the cutting mechanism, surface roughness, cutting force, and workpiece removal rate by micro-milling ZrO_2_ ceramics with a diamond-coated endmill. It was reported that the delamination of the diamond coating was significantly affected by the feed rate, and that the NCD coating lowered the hardness compared to the conventional diamond coating, and it tended to be delaminated faster. Additionally, it was suggested that the lifespan and surface quality improved by maintaining the ductile-machining mode of tools, but premature delamination limited its application in micro-milling of ceramics. Skordaris [7], Hodroj [8], and Lu [9] reported on effects used to improve the interfacial fatigue strength and wear resistance of NCD coatings, such as the use of films annealed in a vacuum [7], tantalum or zirconium nitride-molybdenum bilayers used as interlayers [8], or the change of the basic properties of NCD coatings by doping boron at various concentrations [9]. Similar to this this, several researchers experimentally assessed the mechanical properties of the diamond using diamond-coated cutting tools or coated specimens, but an in-depth analysis of the impact of coating thickness has yet to be performed.

Therefore, in this study, the impact of the coating thickness of NCD-coated ball endmills on their cutting performance was experimentally analyzed. To this end, hot filament CVD (HF-CVD) was applied as 8 and 12 μm NCD coatings on a ball endmill with a 3 mm diameter to perform the plane milling. Then, the correlations between the following were analyzed: the coating surface structure, the abrasion mechanism of the coating and tool, and the machined surface roughness. Correlation analysis of these factors was performed based on the following hypotheses. It was determined that a difference may occur in the macroscopic structure of the coating surface due to the deposition environment that varied depending on the coating thickness. This caused a difference in the abrasion mechanism in the local contact area between the NCD-coated tool and the machining surface and will affect the workability and lifespan of the ball endmill. The results presented in this paper can provide directions for the selection of the suitable surface structure of NCD coating for precision cutting and setting of the coating thickness.

## 2. Materials and Methods

**Figure 1:**
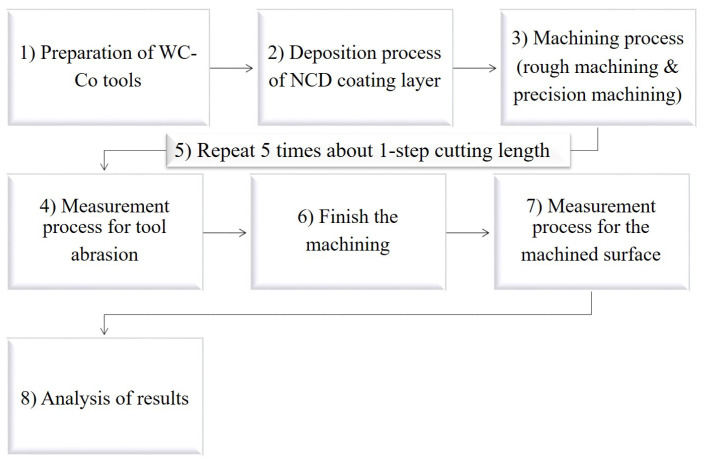
Sequence diagram of experiment of ball endmill for the lifespan and machinability by NCD coating thickness.

The process for analyzing the effect of changes in NCD coating thickness on the lifespan of the ball endmill and the quality of the machined surface is shown in the diagram in Figure 1 and briefly described as follows. (1) After preparing several tungsten carbide-cobalt (WC-Co) ball endmills with an identical shape, (2) Nano coating was performed with coatings of 8 μm and 12 μm, respectively. (3) Using two coated ball endmills, rough machining and precision machining were performed for a cutting length, corresponding to one step under single cutting conditions. (4) The tool was removed, and the abrasion of the cutting edge was observed under an optical microscope. Thereafter, (5) the tool was remounted, and the same machining process was repeated 5 times. (6) After the machining process was finished, (7) the roughness of the machined surface was measured with a surface roughness meter. (8) Finally, an analysis of the results was performed.

### 2.1. Graphite for Glass Mold

Owing to its low thermal expansion, good resistance to thermal shock, and chemical stability, graphite has been used as a crucible since the 15th century [10]. Graphite has a crystal grid of either hexagonal or rhombohedral structure, and the rhombohedral structure is characterized by transforming into a more stable hexagonal structure at temperatures of 1300 °C and higher. Thus, graphite is stable in temperatures of 1000 °C and higher; therefore, as of recently, it is being widely utilized as a mold material for hot compression molding of glass. Among various types of graphite, grade 5 (G5) graphite is fabricated into particles of a few μm or less, unlike general graphite, and it has high strength and hardness, which makes it favorable for surface abrasion and transformation according to the force applied to the mold during hot compression molding [3]. However, microparticles or hard fragments are crushed and ejected instead of cutting chips during mechanical processing using cutting tools. The ejected graphite particles significantly affect the machined surface and damage the cutting tool. Therefore, G5 graphite is one of the most difficult materials for precision cutting [11]. This study analyzes the change in the machinability of the ball endmill in terms of coating abrasion for different thickness values of NCD coating on high-hardness graphite mold material. Accordingly, grade 5 graphite (G5, MERSEN, Bay City, MI, USA) material, which is widely used in commercial glass thermoforming, was selected as the cutting material. The major specifications of this material, as offered by the manufacturer, are summarized in Table 1.

### 2.2. NCD-Coated Ball Endmills

In this study, tool lifespan and change in cutting performance were analyzed based on the following: our previous research [3] and according to the change in thickness of the layer of NCD coating, as well as the coating abrasion of the tool when applied for rough milling and finish milling. As mentioned in Section 1, the coating thickness varied with respect to the radius of the cutting edge, the dimensional accuracy of the tool, and the roughness of the flank and rake surfaces to study its impact on the machining performance of the tool. The change in tool abrasion and coating delamination according to the coating layer thickness also impacted tool lifespan and quality of the machining surface. Therefore, a detailed analysis on the variable that most impacts the degradation of machining performance and the lifespan of the NCD-coated ball endmill was necessary. Tools with identical geometrical data as those used by Lee et al. (our previous research) [3] were selected. These tools were coated with nano-diamond crystals at 8 and 12 μm deposition thicknesses, and the experiment was performed. Detailed information on the tools is provided in Table 2.

The base material of the NCD-coated ball endmill was tungsten carbide–cobalt (WC-Co) material with cobalt content of 6 wt%. As mentioned in Section 1, the WC-Co material was suitable as base material for diamond coating tools because it favored the nucleation for the deposition of the diamond-coating layer [1,2]. In addition, it was used as base material for cutting tools that were easily damaged because it had a hard ceramic (WC) grain on the soft metal (Co) matrix. The mechanical properties of the WC-Co material could be moderated by changing the grain size of the tungsten carbide and the content of cobalt as the binder [12,13]. In general, the mechanical properties of the cutting tool were secured by reducing the cobalt content and fine-tuning the WC grain size. Specifications of WC-Co used as the base material, as provided by the tool manufacturer, are presented in Table 3.

**Figure 2:**
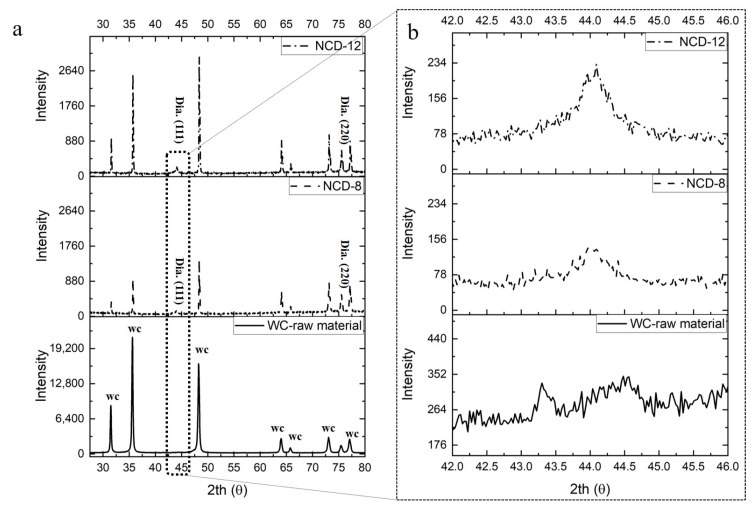
XRD analysis results: (**a**) tungsten carbide (WC) and diamond coating and (**b**) full width at half maximum at 44° diffraction angle.

For NCD-coating, an HF-CVD method was used to deposit the ball endmill with 8 μm and 12 μm coatings thickness, respectively. The BALDIA NANO coating applied in this study was primarily recommended by Oerlikon Balzers for processing carbon-fiber-reinforced polymers (CFRP) or ceramic materials. This implied that the coating layer was known to exhibit the best performance to the recommended materials and that this machining performance may be slightly lowered in the case of graphite. However, this study compared the relative tool lifespan for each coating thickness and machining performance. Thus, NCD coating from Oerlikon Balzers (one of the major coating manufacturers) was applied. The appearance of nano-diamond crystal grains in the fabricated ball endmill could be identified through X-ray diffraction (XRD) analysis. Figure 2a illustrates the XRD pattern of NCD coating for each coating thickness. NCD coating was characterized by the occurrence of full width at half-maximum properties at a diffraction angle of 44°, which corresponded to the (111) plane of the cubic diamond, as shown in Figure 2b [14,15].

### 2.3. Machine Tools and Milling Conditions

It is critical to optimize the cutting conditions and select suitable cutting tools because of the high hardness and brittleness of the graphite material for glass thermoforming. This is because local fractures are formed due to brittleness, the fragments thus create a flow along the cutting edge or continuously collide to damage and deform the cutting tool. Additionally, because the use of lubricant can cause the graphite particles to fuse to the workpiece and the tool surface, machining is performed in a dry environment. The machining system in this research uses a 3-axis CNC high speed machine tool (Roeders, RXP500, Soltau, Germany), identical to that used in the our previous research, which was also referenced for the recommended cutting conditions provided by the tool manufacturer [3]. The major specifications of the machine tool and the cutting conditions are listed in Table 4 and Table 5.

**Figure 3:**
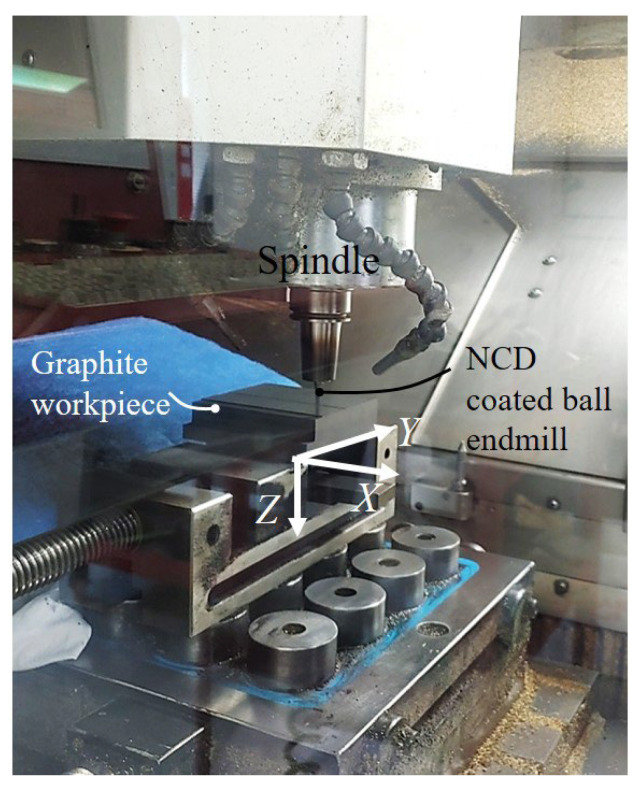
Experimental set-up of workpiece and NCD coated ball endmill.

As shown in Figure 3, the diamond-coated ball endmill was equipped on the spindle, and the graphite workpiece was fixed to the vise of the XY-axis feed stage. As mentioned earlier, this research aims to analyze impact of the thickness of the coating on the abrasion and the machining performance of the ball endmill. Therefore, the cutting condition was fixed, the abrasion of the tool was measured for each certain cutting length, and, subsequently, the variation in roughness of machined surface was analyzed.

**Figure 4:**
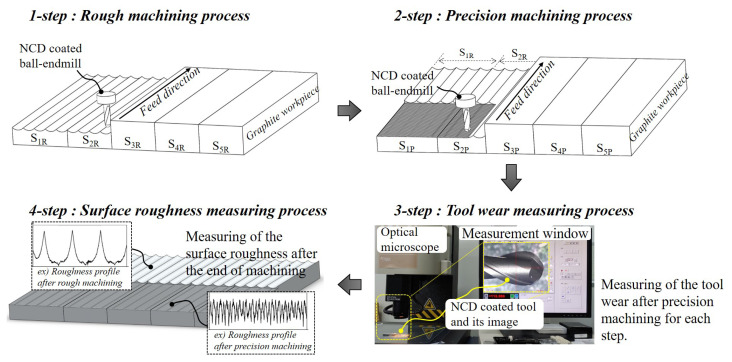
Procedure of the milling processes, tool abrasion and machined surface roughness measurement.

Figure 4 illustrates the machining and measurement order. Rough machining was performed with a ball endmill for cutting length of one step, and precision machining was performed for the area that corresponds to half of the rough-cut area. Subsequently, machining was stopped, the ball endmill was removed from the spindle, and the abrasion of the cutting edge was measured using an optical microscope. After the measurement, the ball endmill was mounted back onto the spindle, and rough machining, precision machining, and tool abrasion measurement was repeated until end of the machining. After machining, the 1164 m length that corresponds to the five-steps cutting length, the surface roughness that had been rough machining and precision machining was measured for each step. The cutting length for each step was 232.8 m, from which the rough machining length was 208.8 m, and the precision machining length was 24 m.

### 2.4. Measurement Systems

The abrasion process of the cutting edge of the ball endmill according to the cutting length was observed using an optical microscope (ROI Instrument, Rochester, NY, USA). A 3D-measuring laser microscope (OLS5100&LEXT5100-SAF; OLYMPUS, Tokyo, Japan) was used for the 3D image of the machined surface and the cutting edge before and after machining. Changes in the roughness of the machined surface were measured using a surface roughness measuring instrument (Nanoscan 855; JENOPTIK, Jena, Germany). The roughness was measured at 3 points per step, and the value was obtained by the centerline averaging method (Ra), and the result was expressed as the average value of Ra. Here, the measurement proceeded perpendicular to the direction of tool transfer. For the generation of nano-diamond crystals, XRD equipment (X’Pert-Pro MPD; Malvern Panalytical, Malvern, UK) was used. For recording the XRD profile, 30 mA and 40 kV CuKα radiation was used. Scanning was performed within the range of 2θ for 20–80° in interval 0.01° at a canning rate of 4° per minute.

## 3. Results and Discussion

### 3.1. Morphology and Surface Roughness of NCD Coated Surface

**Figure 5:**
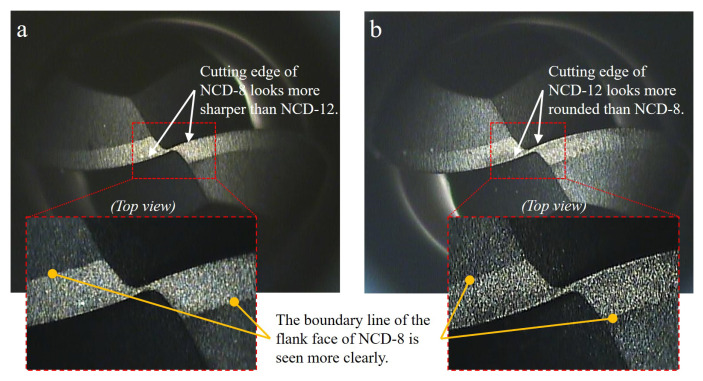
Optical microscope images of NCD coated ball endmill with different coating thickness: (**a**) 8 μm and (**b**) 12 μm.

**Figure 6:**
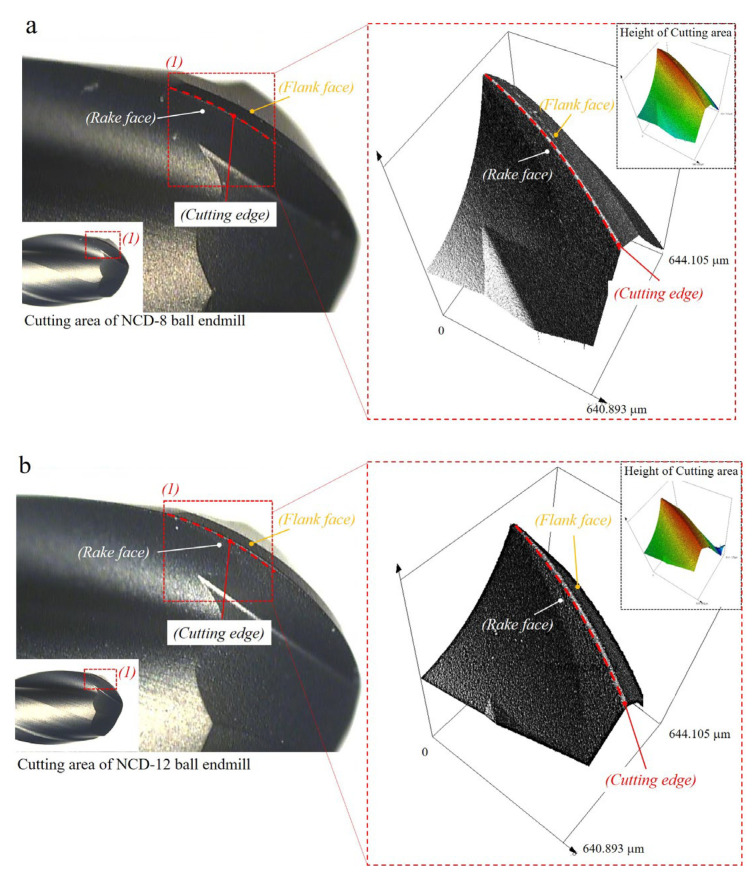

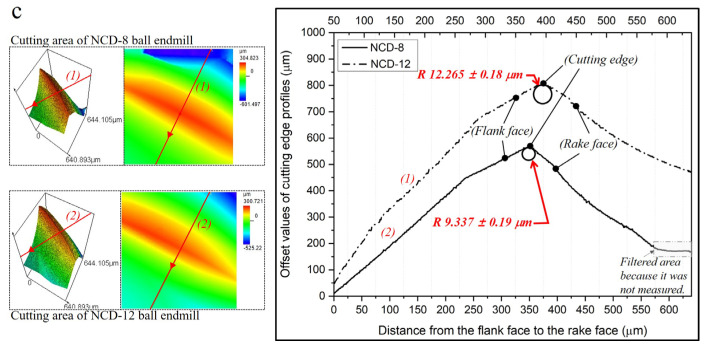
Information of cutting area for two different NCD coating tools: Optical and 3D scan images of (**a**) NCD−8 and (**b**) NCD−12. (**c**) Radius profiles of cutting edges for NCD−8 and NCD−12.

To analyze the effect of increasing the NCD coating thickness on tool abrasion and machinability, the coating surface and the change in the cutting-edge radius of the ball endmill according to the coating thickness were observed before the machining tests. An optical microscope and a 3D confocal laser microscope were used to observe and analyze the coating surface. Figure 5a,b are the optical microscope images at 76 and 148 times magnification (inside red dot of box). In the 8 μm-thick NCD coating (Figure 5a), compared to its 12 μm-thick counterpart (Figure 5b), the boundary of the cutting face from the endmill is more defined and the cutting edge is relatively sharper. This can be identified through the measurement of the cutting edge curvature. Figure 6 shows the measured results of the radius of the cutting edge using the analysis tool after 3D scanning, which was performed using a 3D confocal laser microscope. Including the measurement uncertainty at the 95% confidence level, the results were R 9.337 ± 0.18 μm and R 12.265 ± 0.19 μm each. Here, the curvatures measured for the 8 μm and 12 μm coating thicknesses were 0.107 ± 0.0018 (1 μm^−1^) and 0.082 ± 0.0012 (1 μm^−1^). Qin et al. [5] and Aslantas et al. [16] reported that the increase in coating thickness or the existence of coating increased the cutting edge radius, and, in this study, identical results were obtained.

**Figure 7:**
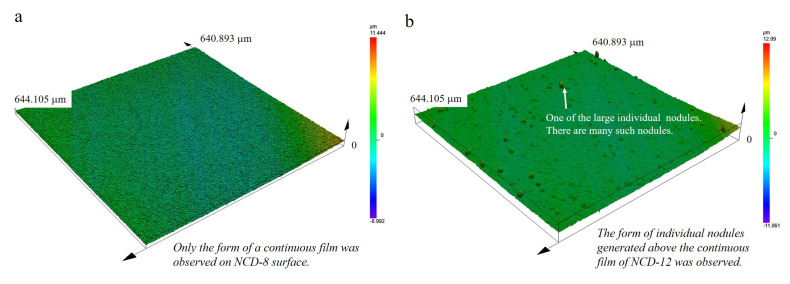

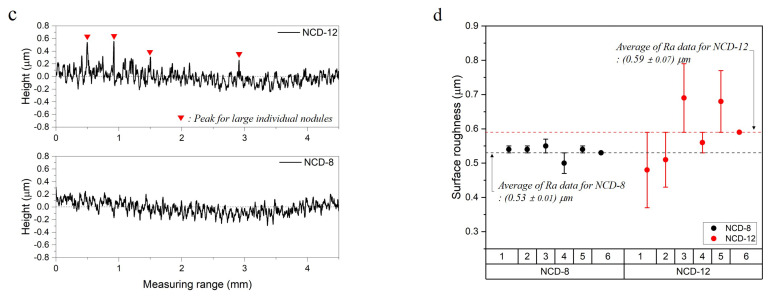
Surface information for each NCD coating thickness: 3D scan image of (**a**) NCD−8 and (**b**) NCD−12. (**c**) Surface roughness profiles of NCD−8 and NCD−12, (**d**) Ra value deviation of NCD−8 and NCD−12.

The surface roughness of the cutting area of tool was another factor that varied according to the coating thickness. The surface was scanned using a 3D confocal laser microscope, and the impact of the coating thickness was analyzed with the centerline average roughness (Ra) data obtained with a contact type surface profile meter. As shown in Figure 7a, individual nodules were uniformly distributed by forming continuous films throughout the surface with an 8 μm thick coating. In contrast, individual spherical or hemispherical nodules that were few μm in size were dispersed throughout the continuous and uniform (NCD-12) surface coated with 12 μm thickness (Figure 7b). This can be confirmed from the surface roughness profile (Figure 7c) and the deviation of Ra value (Figure 7d). In the surface profile of the NCD-12 sample, high peaks (indicated as red inverse triangles) appeared intermittently, and the range of Ra deviation of the two samples was −0.03–0.02 and −0.09–0.11. Here, the average Ra values were confirmed to be 0.53 ± 0.01 μm and 0.59 ± 0.07 μm, respectively, including measurement uncertainty when measured at the 95% confidence level. From this result, the individual nodules collected in one or 2–3 concentrations according to the coating thickness were distributed and developed on the continuously combined nodules to change the surface roughness.

The sporadic stacking of the individual nodules that affects the surface roughness could be impacted by various factors such as the active gas, its concentration, the pretreatment method, and the deposition method because it is a key process variable that controls the nucleation and growth of the diamond [17,18]. Zhang et al. [17] reported that the film properties of HF-CVD diamond were greatly affected by deposition parameters such as substrate temperature, total pressure, and carbon concentration. He studied the effects of these three variables and confirmed that the substrate temperature and total pressure affected the crystal morphology and growth rate of the diamond film. Haubner et al. [18] reported that early studies for growing diamond nuclei in the gas phase investigated the function of atomic hydrogen, various growth methods, and process parameters. It was also stated that the studies included analyses of the interactions between substrates such as cemented carbide and ceramics and the deposition gas atmosphere. However, there has been no accurate report on which factor among the process controls variables for increasing the deposition thickness creating the secondary structure on top of the uniformly and continuously combined primary coating layer. Thus, we analyzed in depth the previous research on the nano-diamond nucleation mechanism and concluded that the individual nuclei on the NCD-12 coating surface were in the process of nuclear growth before forming a continuous film [19,20,21,22].

**Figure 8:**
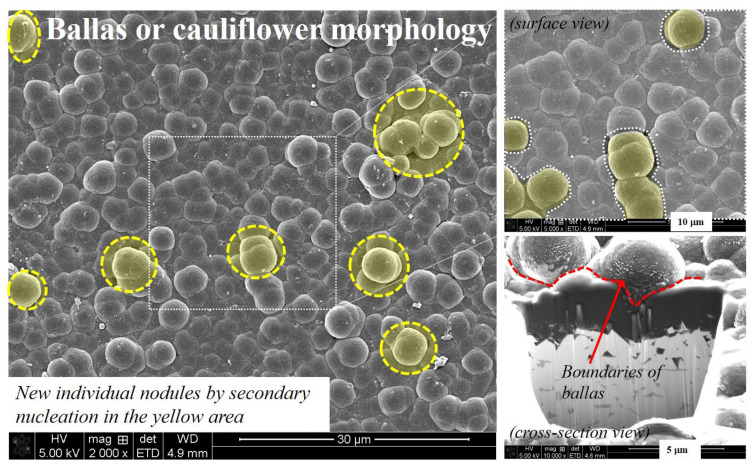
Example SEM images of the NCD coating surface (coating thickness: 10 μm), and the distribution of individual nodules generated by secondary nucleation.

According to Kulisch et al. [19], NCD coating forms a “ballas” or “cauliflower-like” form as the individual spherical nodules increase with increasing deposition time, grow radially, and, finally, merge. According Kulisch et al. [19] and Lux et al. [20], this is due to the low primary nucleation density and high secondary nuclear growth rate of NCD. The nucleation density of the diamond in most substrates is significantly low due to its high surface energy. Therefore, individual crystals are primarily sporadically created on the pre-processed substrate. Eventually, as these individual nodules grow thicker than the nodule diameter due to the high secondary nuclear growth rate of NCD, they merge as microtwin structures and form a continuous film (see white area of surface view in Figure 8). According to Barbosa et al. [21] and Williams et al. [22], the generated diamond film is mainly composed of SP^3^ carbon, but may contain non-diamond carbon composed of several bonding configurations (SP, SP^2^) and disordered carbon depending on the deposition environment. In the case of the nanoballas’ structure, grain boundaries of non-diamond carbon surround the nanograins of SP^3^ carbon, and, in the case of the cauliflower structure, it has been described that it is more similar to columnar microcrystalline diamond. Additionally, these non-diamond carbons facilitate the regeneration of nanonuclei at the boundary.

An example of the NCD-coated surface showing a similar structure to that described above is shown in Figure 8. NCD was deposited with a thickness of 10 μm on the same tool used in this study, and it could be confirmed that the nodules of few μm in size had distinct boundaries and were connected to each other. In addition, individual nodules were formed on top of the connected grains. Through this, it seemed that the rate of the nuclei that newly grew on of the ballas structure increased as the deposition progressed in the 12 μm thickness coating compared to its 8 μm counterpart. It should be noted that the increased surface roughness due to the individual nodules that were newly formed onto the continuous nodules could affect the tool abrasion and machined surface. This was because the individual nodules that appeared as microscopic protrusions on the cutting-edge surface could damage the machining surface or affect the coating abrasion behavior.

### 3.2. Tool Wear According to the Coating Thickness of NCD Ball Endmills

**Figure 9:**
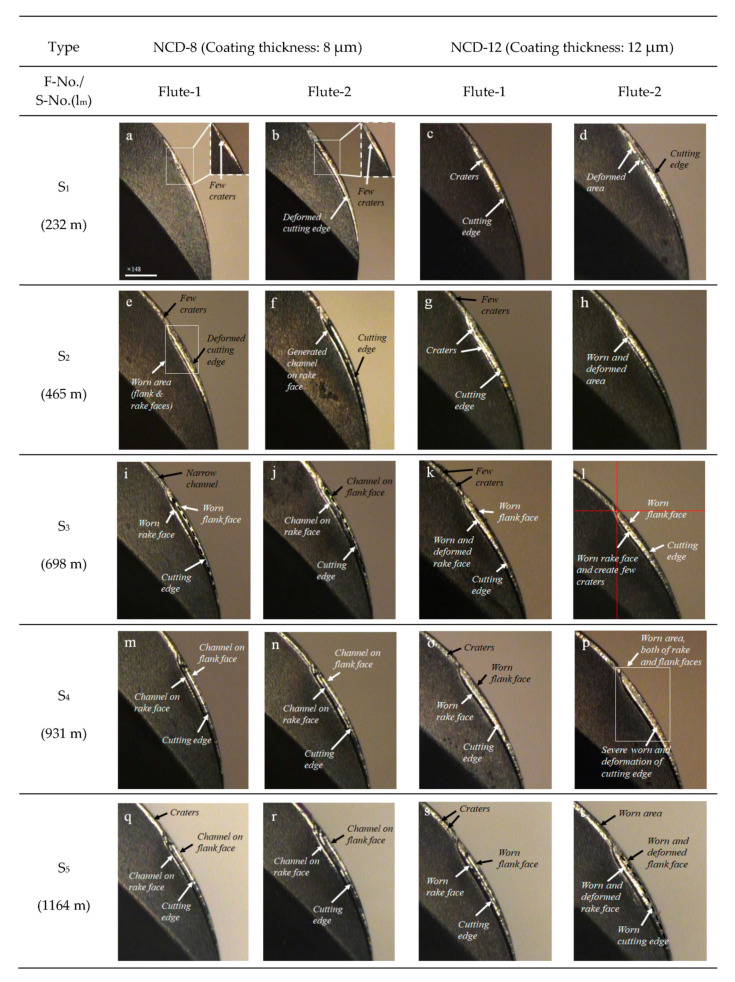
Flank and rake abrasion change in NCD tool according to cut length for each coating thickness: (**a**–**d**) S_1_, (**e**–**h**) S_2_, (**i**–**l**) S_3_, (**m**–**p**) S_4_, (**q**–**t**) S_5_.

**Figure 10:**
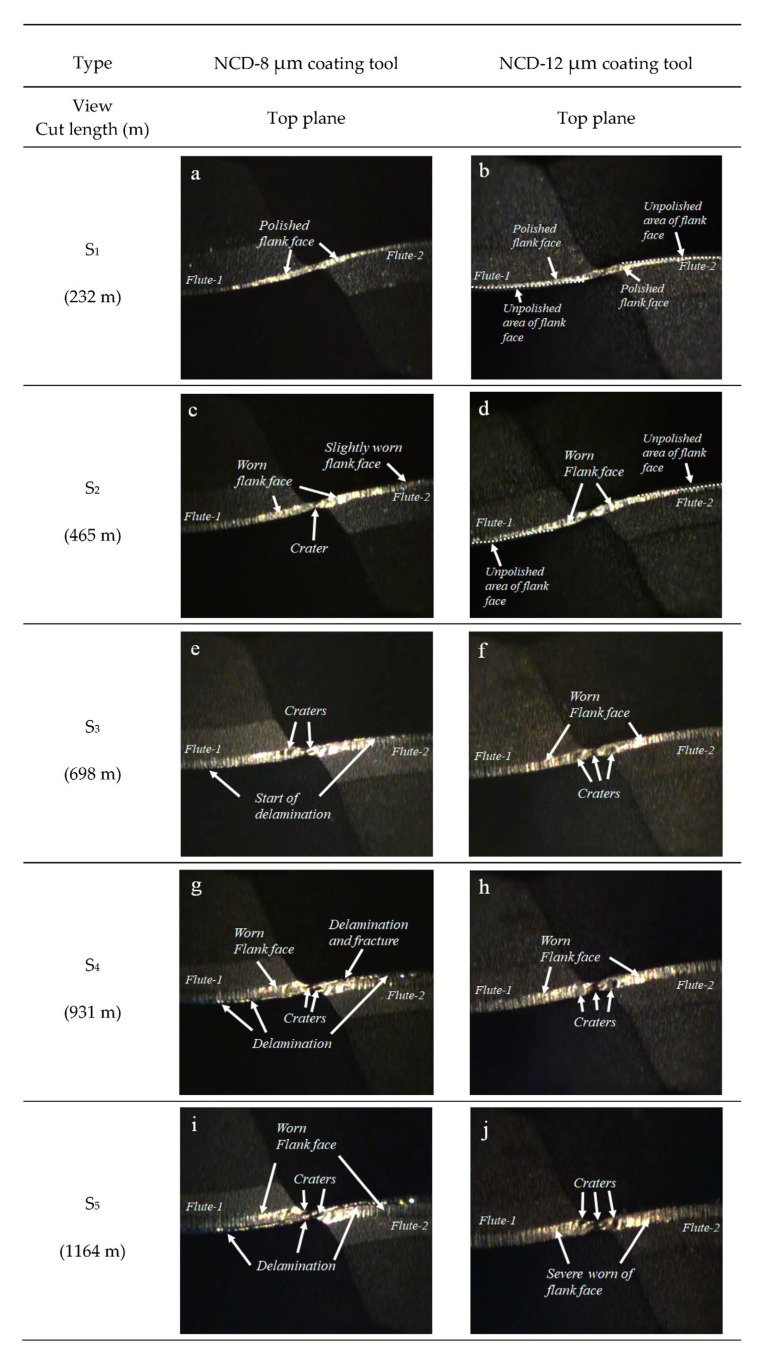
Flank and rake abrasions observed using the top views of NCD−8 μm and NCD−12 μm coated tools according to cut length: (**a**,**b**) S_1_, (**c**,**d**) S_2_, (**e**,**f**) S_3_, (**g**,**h**) S_4_, (**i**,**j**) S_5_.

The abrasion regarding the cutting length of the ball endmill was observed for specimens with two different coating thicknesses, 8 and 12 μm, and the results are illustrated in Figure 9 and Figure 10. As shown in Figure 9, the coating abrasion on the flank and rake faces of the cutting edge of ball endmill with two different coating thicknesses progressed in a similar manner, while the extent of abrasion differed. The damage to the diamond coating during graphite processing in our previous research [3] initiated with the polishing of the coated surface. As identified in the S_1_ stage in Figure 9, in this study, we observed small deformations of the cutting edge, along with the polished surface and the formation of craters on the rake face. Furthermore, an identical channel formed on the rake face was also formed on the flank face accompanied by a significant deformation in the cutting edge, and the abrasion gradually expanded toward the tool shank.

As described above, the final abrasion of the diamond-coated tool in graphite machining is in the form of long valleys created by delamination on both sides of the flank and rake faces with the deformed cutting edge in between. In this study, the width expansion of the abrasion in the 8 μm coating after S_2_ was slow, while a long channel of the same shape had formed on the two cutting faces. In the 12 μm thickness specimen, crater abrasions were observed initially, and the abrasion width rapidly broadened, which followed by a severe deformation of the cutting edge after step S_3_. However, no long channel was observed due to delamination.

**Figure 11:**
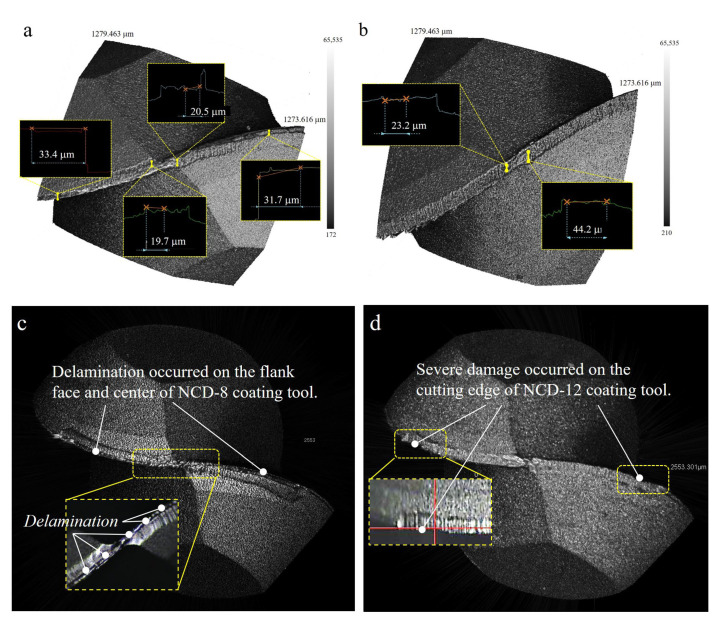
Tool edge and free plane abrasion comparison according to NCD coating thickness: (**a**,**c**) 8 μm thickness, and (**b**,**d**) 12 μm thickness.

The difference in abrasion behavior according to the coating thickness was more clearly defined in the tool tip measurement image. For each cutting length, Figure 10 illustrates the abrasion on the flank face and tool tip where the cutting edge intersects. The 3D scan image of the tool tip abrasion after machining is presented in Figure 11. As the cutting length increased, the major abrasions of the two ball endmills with different coating thicknesses showed the following differences: In the case of 8 μm coating thickness, delamination of the flank face was dominant, whereas in the 12 μm coating thickness, the crater abrasion at the tool tip was identified as the major abrasion, and no delamination was observed on the flank face, as shown in the side view. Minor craters were discovered from the cutting length of 465 m in the case of the 8 μm NCD coating thickness; however, the average crater width after machining was 20 μm as delamination progressed to the tool tip, which was smaller than that observed for the 12 μm coating endmill, which exhibited an average crater width of 34 μm (Figure 11).

### 3.3. Change in Machined Surface Roughness with Respect to Flank Abrasion

**Figure 12:**
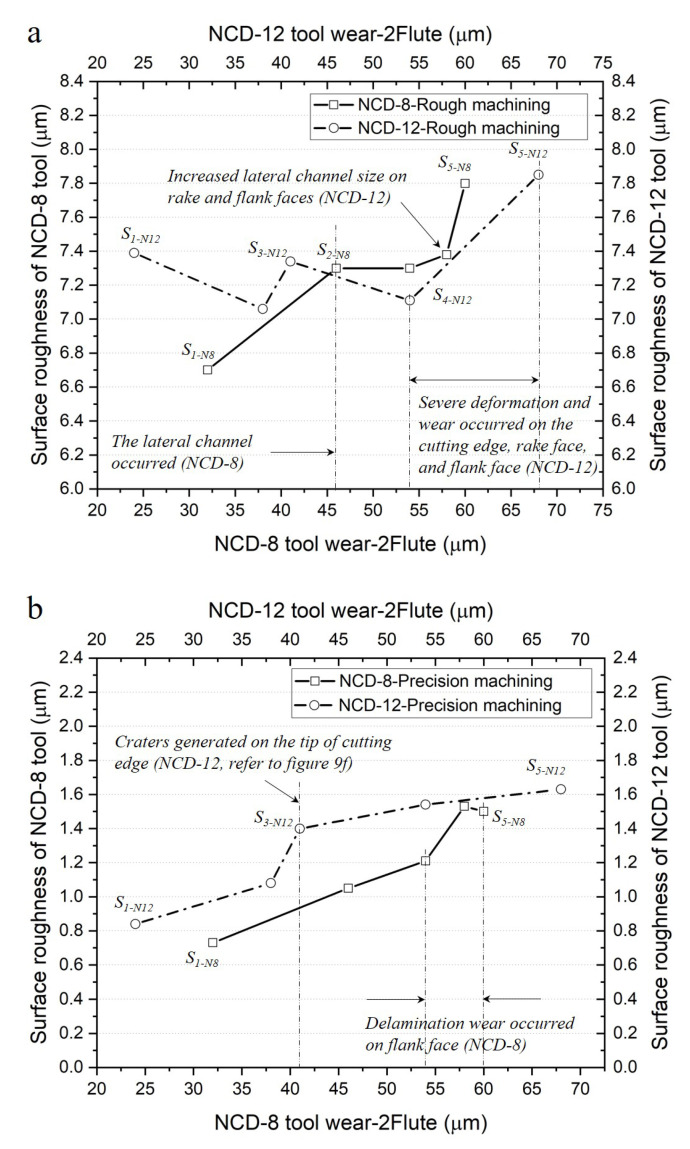
Comparison of change in surface roughness with respect to ball endmill abrasion according to coating thickness and machining conditions: (**a**) rough machining and (**b**) precision machining.

In machining with the ball endmill, the cutting area varied according to two cutting depths in the direction of the tool axis and in the direction the tool radius. This is because the cutting edge was generated along hemispherical shape. Therefore, the side (flank and rake faces) of the cutting edge was involved in the rough machining that cut relatively significantly in the axial direction, and precision machining was performed on the tool tip and at the side close to the axis of rotation. Figure 12 graphs the change in machined surface roughness with respect to the flank face abrasion of the ball endmill for a total cutting length of 1164 m.

In rough machining, the initial surface roughness from the 12 μm coating endmill was higher by approximately 10% than that of the 8 μm coating, despite the abrasion width being smaller in the flank face. After the delamination from the 8 μm coating endmill (S_2_), the roughness of the machined surface had reversed, but the surface roughness of the 12 μm coated endmill machining rapidly increased again after S_4_, and similar roughness values were observed in both cases after the machining was complete. In precision machining, the surface roughness of the 12 μm coated ball endmill machining continuously remained higher than that of the 8 μm coated ball endmill machining over the entire cutting length. The machined surface roughness rapidly increased between S_2_ and S_3,_ where crater abrasion occurred at the tool tip. The machined surface roughness of the 8 μm coated ball endmill rapidly increased between S_3_ and S_4_, where the occurrence of coating delamination initiated in the flank face.

### 3.4. Impact of Coating Thickness on Tool Abrasion and Machined Surface Roughness

The impact of the NCD coating thickness on the ball endmill abrasion and roughness of the machined surface were analyzed. The analysis of the abrasion tendency of the side and tip of the cutting edge showed that the abrasion of the cutting edge due to the coating thickness difference exhibited different patterns at the tool tip and on its side. In the case of machined surface roughness, it was observed that a thicker coating was more favorable for rough machining, and a thinner coating thickness was more favorable for precision machining. The impact of NCD coating thickness for the results of this study was analyzed relevant to the following two facts: First, the change in coating thickness affected the delamination abrasion at the interface; thus, a difference in abrasive behavior was observed at the flank and rake faces and the tool tip. Second, the variation in coating thickness affected the surface roughness of coating layer, which further altered the machining performance of the two NCD-coated ball endmills.

In general, it is known that the stronger the adhesion between the interfaces the longer the tool’s lifespan is, as the coating thickness increases. Through turning machining experiments of the insert tip coated with various thicknesses of nano-diamonds, Qin [5] found that the fastest delamination occurred in the case of lower coating thickness of 4 μm and reported that the tool abrasion and beginning of coating delamination varied according to the coating thickness. This was attributed to the elongated tool lifespan before delamination due to the increase in cutting load capacity as the coating thickness increased. Thus, thicker coatings were unfavorable for brittle fractures due to crack propagation from the high tensile strength of the substrate, but the critical load for delamination at the interface increased due to delayed initiation of plastic yielding. In our previous research, delamination had also been delayed through the micro scratch test in the MCD coating ball endmill with high adhesion strength between the interface [3]. Through this, it had been identified that the increase in coating thickness delayed the beginning of the delamination of the coating and affected the tool’s lifespan before the coating delamination started.

The previous sections discussed how the tool abrasion and machined surface were affected by the roughness of the cutting edge surface due to the newly generated individual nodules on the continuous nodules. In general, abrasion damaged the opposite surface due to the asperity that existed on the contact interface of friction pair and occurred as the damaged asperity was detached by various mechanisms [23]. As the NCD coating thickness increased, the individual nodules generated on the cutting surface of tool could act as irregularities. This unevenness had a higher hardness than the opposite material, which, in this study, was graphite. Therefore, it damaged the graphite surface and because it is made of diamond crystals, i.e., a carbon aggregate, it can cause intergranular adhesion. The NCD coating layer was also damaged due to repeated collisions with the graphite workpiece and the abrasive particles.

**Figure 13:**
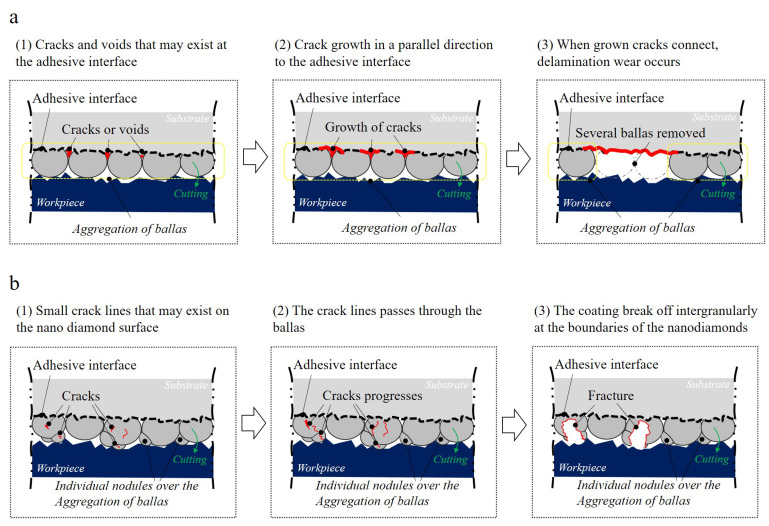
Nano-diamond balls abrasion and processed surface abrasion mechanism.

According to Hamzah [24], damage to NCD coating either occurs (1) along the boundary of the ballas aggregate or (2) intergranularly at the nano-diamond boundary of the ballas aggregate. Therefore, delamination abrasion that includes several ballas occurs at the interface between the coating and substrate as the damage progresses according to the former case. In the latter case, damage with a high gradient, similar to the crater, is formed in the inward direction of the substrate due to the crack that progresses inwards the ballas along the nano-diamond surface (Refer to Figure 13). The author attributes this to the bonding force the nano-diamond being stronger than the adhesive force between the diamond ballas and the substrate. Thus, the surface roughness of the cutting edge, which increased due to the coating thickness, affected the machined surface, as shown in Figure 13.

First, the hard individual nodules, which increased the surface roughness, intensified the graphite surface damage during initial processing and degraded the roughness of the surface processed with a 12 μm coated ball endmill. Second, the damage of the individual nodules that affected the roughness induced the damage on the flank and rake faces of the 12 μm coated ball endmill, which rapidly led to the increase in the roughness of the machined surface. The lateral damage had a similar form to the crater abrasion of the insert edge, and the crater abrasion that occurred concavely due to the friction with the cutting chip on the upper surface at some distance from the tool nose, which intensified as the roughness of the cutting surface increased. Third, the reason for the dominant occurrence of delamination abrasion from the 8 μm coated ball endmill was because the damage to the coating can occurred along the boundary of the ballas aggregate that usually formed a continuous film.

## 4. Conclusions

The application of coating in cutting tools increases the durability of abrasion and improves the machining performance and tool lifespan. Thus, the use of coated cutting tools is gradually expanding to hard-to-machining materials such as CFRP, considerably hard graphite, and ceramics. However, because the cutting force changes as the radius of the cutting edge and surface roughness increases, it is necessary to analyze the impact of the coating thickness. In this study, the abrasion mechanism, lifespan, and machining performance of the NCD-coated ball endmill were analyzed according to the coating thickness. Thereby, we aimed to present a direction toward improving the NCD coating tool. The results of this study were as follows.

- The change in coating thickness affected the formation of the nano crystal structure, and the formation of individual nodules on the top of the continuous film increased the coating surface roughness.
- The increase in coating surface roughness, ultimately, resulted in the degradation of the machined surface roughness, damage to the 12 μm-coated cutting edge, and crater abrasion.
- Delamination abrasion dominantly occurred in the 8 μm coated ball endmill, but the machined surface had a higher quality in the 8 μm coated ball endmill than in the case of its 12 μm counterpart in precision machining.
- Because of delayed delamination, the 12 μm coated ball endmill did not have coating delamination at the flank and rake faces.

As a result, it was confirmed that the increase in the lifespan of the NCD coating was not related to the improvement of the machined surface quality. The improvement of the machined surface quality was influenced more by the roughness due to the change in the coating surface structure than by the effect of delaying the abrasion of the tool due to the increase in the coating thickness. Considering the functions of NCD coating tools, which are generally applied to the precise cutting of difficult-to-cut materials, it could be seen that the optimum control of coating thickness and coating surface roughness was necessary to increase the lifespan and simultaneously secure precision machinability. Therefore, for this purpose, the deposition parameters must have been precisely controlled, and it can be applied to precision machining of CFRP materials for automobile weight reduction and precision machining of glass molds for manufacturing mobile displays with a curvature of 180° if the optimal coating thickness is derived.

In this paper, the study on cutting conditions is limited by analyzing the effect of NCD coating thickness on only one cutting condition. Optimization of the coating thickness requires consideration of various cutting conditions such as cutting speed, feed rate, and depth of cut. In addition, research is needed to analyze the impact of the change in cutting edge radius and cutting force according to the coating thickness on the machining surface and coating abrasion. It is also required to derive the optimal coating thickness by broadening the range of coating thickness values. Through this, it is necessary to derive the optimal cutting conditions for each coating thickness, and to present the optimal coating thickness and recommended cutting conditions for the field where the NCD-coated ball endmill is applied by linking it with the tool production cost, material removal rate, and machining accuracy.

## Figures and Tables

**Table 1:** Specifications of the G5 mold material used in this study (Mersen datasheet).

Properties	Typical Values
Bulk Density (g/cm^3^)	1.84
Grain Size (μm)	7
Flexural Strength (MPa)	72
Tensile Strength (MPa)	43
Compressive Strength (MPa)	148
Hardness Rockwell “H”	90
Coefficient of Thermal Expansion (400 to 500 °C)	5.7 × 10^−6^/°C
Thermal Conductivity	102 W/m°C

**Table 2:** Information of Tool geometry and parameters.

Tool Geometry	Tool Parameters	Values
** 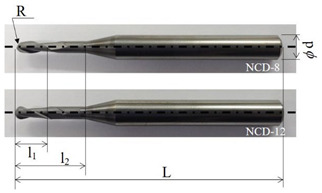 **	Radius, R (mm)	1.5
	Diameter of tool shank, d (mm)	6.0
	Length of cut, l_1_ (mm)	8.0
	Effective Length, l_2_ (mm)	16.0
	Length of tool, L (mm)	60.0
	Helix angle, θ (°)	30
	Flute number, z	2

**Table 3:** Characteristics of WC-Co cutting tool material (Grade: GK05A).

Grain Size (μm)	Cobalt (%)	Density (g/cm^3^)	Hardness (HV_30_)	Fracture Toughness (MPa m^1/2^)
1.0	6.0	14.9 ± 0.1	1740 ± 50	<8.9

**Table 4:** Specifications of machine tools.

Specifications	Values
Travel distance (mm)	600 × 450 × 400
Spindle speed N (rpm)	0~42,000
Max. Feed rate F (mm/min)	60,000
Machining accuracy (μm)	±2
Diameter of cutting tools (mm)	0.2~12
Max. load on table (kg)	500

**Table 5:** Milling conditions.

Milling Parameters	Values
Cutting velocity (m/min)	104
Spindle speed (rpm)	11,000
Feed rate (mm/min)	1200
Feed per tooth (mm/tooth)	0.055
Axial cut depth (mm)	0.3
Radial cut depth (mm)	0.9 (rough machining)/0.1 (precision machining)
Total cutting length (m)	1044 (rough machining)/120 (precision machining)
Lubrication environment	Dry

## Data Availability

Not applicable.
